# Physicians’ attitudes and perceptions toward the integration of artificial intelligence into pediatric hematology–oncology in Saudi Arabia

**DOI:** 10.3389/fmed.2026.1820831

**Published:** 2026-07-03

**Authors:** Sawsan Alblewi

**Affiliations:** Department of Pediatrics, Faculty of Medicine, University of Tabuk, Tabuk, Saudi Arabia

**Keywords:** artificial intelligence, attitudes, hematology, oncology, physicians, Saudi Arabia

## Abstract

**Background:**

With increasing interest in integrating artificialPrevalence of nasal myiasis in slaughtered camels intelligence (AI) into healthcare in Saudi Arabia, many benefits of using AI for data analysis, pattern recognition, and early disease detection are being recognized by many physicians practicing pediatric hematology–oncology in the country. However, there are concerns regarding AI’s limitations, ethical issues, and potential job displacement. This study aimed to explore the attitudes and perceptions of physicians practicing pediatric hematology–oncology in Saudi Arabia toward the use of AI.

**Methods:**

A cross-sectional study was conducted in Tabuk, Saudi Arabia, and included 132 medical doctors. An online questionnaire comprising several structured questions was used for data collection.

**Results:**

This study found that 74.2% of physicians had a positive view of the integration of AI into their clinical practice. Physicians’ professional title (*p* = 0.011) and years of work experience (*p* = 0.019) were significantly related to their views. The main advantages of using AI, as reported by 74.2% of participating physicians, included efficiency, accuracy, and cost-effectiveness. Other advantages and disadvantages were mentioned by 12.1 and 71.9% of participants, respectively. The main challenges identified were privacy, interpretability, and ethical concerns. However, 56.8% of the participating physicians believed that human physicians should remain the primary decision-makers regarding patient treatment when using AI. The majority of the participating physicians recognized the potential of AI in the diagnosis and treatment of patients.

**Conclusion:**

Attitudes toward AI among pediatric hematology–oncology physicians are overwhelmingly positive, as they recognize AI’s potential to be an efficient and accurate tool in clinical practice. The vast majority of these physicians consider AI to be a complement to human capabilities and emphasize the need for physicians to oversee AI-assisted clinical decisions.

## 1 Introduction

Artificial intelligence (AI) is rapidly changing many aspects of life. Within the healthcare system, many new AI-driven technologies and applications are currently in use for clinical decision-making, prediction, and a host of other functions that could improve patient outcomes. There is also potential for many positive changes for healthcare professionals, including reduced costs and workload (1).

The use of AI in healthcare in Saudi Arabia is becoming increasingly important and is gaining the interest of many physicians. While they appreciate the ability of AI to process large amounts of data, identify patterns and trends, and facilitate the early diagnosis of diseases, there are several limitations of AI, ethical concerns, and fears of being replaced by AI (2). Some physicians worry that AI could undermine their clinical independence, while others believe that well-implemented AI will enhance their clinical work by serving as a powerful tool to support their clinical decision-making and practice (3–5). In addition to their potential effects on patient safety and quality of care, some doctors worry about how AI could affect patient autonomy and the doctor–patient relationship (6). Physician acceptance of AI largely depends on adequate training and a clear framework defining the role of AI in clinical practice to build trust in AI systems (7).

The pediatric hematology–oncology discipline addresses complex and life-threatening diseases such as leukemia, lymphoma, and other blood disorders. In this specialized field, AI can provide valuable support in addressing current challenges faced by healthcare providers, including the increased need for early and accurate diagnosis, the implementation of precision medicine, and the optimization of treatment strategies to improve patient survival and quality of life (8).

Accurate diagnosis is one of the key roles of AI in hematology–oncology. Traditionally, the diagnosis of complex blood malignancies involves the interpretation of pathology, molecular, and imaging data. AI-driven algorithms have shown notable accuracy in interpreting radiological scans, bone marrow biopsies, and blood smears, thereby reducing human error and facilitating the identification of early disease indicators (9). Furthermore, genetic and molecular differences in hematologic malignancies can result in diverse responses to therapy. AI has shown a great capacity to enhance tailored therapy, with AI systems predicting which treatments are most likely to be effective for specific patients (10). With respect to predictive analytics and prognosis in hematology–oncology, machine learning models powered by AI have shown high efficiency in predicting disease progression and treatment success (11).

Although AI has shown potential in pediatric hematology–oncology, its successful incorporation into clinical practice is primarily dependent on the attitudes and perspectives of physicians, who play a crucial role in promoting its use. To create AI applications that meet the needs of physicians and guarantee their successful implementation in pediatric hematology–oncology, it is imperative to understand these perspectives. This study aimed to understand the views and opinions of pediatric hematology–oncology physicians in Saudi Arabia regarding the use of AI in their clinical practice.

## 2 Materials and methods

### 2.1 Study design

This cross-sectional study was conducted in Tabuk, Saudi Arabia, between 1 December 2024 and 31 January 2025. This study received approval from the Research Ethics Committee at the University of Tabuk, Tabuk, Saudi Arabia. To maintain data confidentiality, each participant was assigned a unique code, and no names or personally identifiable information were collected. Prior to participation, informed consent was obtained from each participant after providing a comprehensive explanation of the study objectives.

### 2.2 Study population

We included physicians specializing in pediatric hematology–oncology in Saudi Arabia who voluntarily agreed to complete the questionnaire. Physicians who did not specialize in pediatric hematology–oncology, those who worked outside Saudi Arabia, and those who refused to participate were excluded from the study. The participants were pediatric hematology–oncology physicians working in different healthcare settings across Saudi Arabia. They had different medical titles and varying levels of experience and exposure to cases. Therefore, the case scenarios they managed were neither identical nor evenly distributed among the participants. Participants were recruited through the Saudi Arabia Pediatric Hematology Oncology Society (SAPHOS) and WhatsApp professional groups using a convenience sampling approach with voluntary participation. The sample size was determined using the Qualtrics calculator, which is based on a total study population of 200 physicians specializing in hematology and oncology, with a 95% confidence level and a 5% margin of error. The required sample size was 132.

### 2.3 Data collection and definition of AI decision support systems

Data were collected via a structured online questionnaire adapted from a previous study (12), with the author’s permission. The questionnaire was reviewed to ensure clarity, relevance, and suitability for pediatric hematology–oncology physicians before distribution. Minor adjustments were made to the questionnaire to better align with the objectives of this study and its target group.

The term “AI decision support systems” in this study refers to a wide array of clinical support applications powered by AI. It encompasses machine learning and predictive analytics for clinical decision support. The study under review does not distinguish between generative AI, imaging AI, and other specialized models and applications.

This physician questionnaire assesses physicians’ general attitudes toward integrating AI into their clinical work for pediatric oncology patients with cancer. It does not require respondents to evaluate specific tools or tasks, such as diagnosis support, treatment planning, tumor board, and relapse management for patient monitoring.

The tool consists of three sections. The first section comprises a questionnaire designed to gather participants’ demographic and professional data. The information includes age, sex, nationality, medical subspecialty, highest professional degree, years of experience, and the practice location of the participants within Saudi Arabia. The second section is a 14-item questionnaire that measures physicians’ attitudes toward the use of AI in clinical settings. The items were scored on a 5-point Likert scale. Items 1–7 and 9–13 were scored on a scale ranging from 1 (‘strongly disagree’) to 5 (‘strongly agree’), and items 8, 11, and 14 were reverse-scored accordingly. A total attitude score of 43 or above indicates a positive attitude. The third section comprises several open-ended questions. These questions aim to explore participants’ experiences using AI in clinical settings, the factors influencing their willingness to implement AI, the problems they encounter during implementation, and their perceptions of the relationship between physicians and AI.

The participants were recruited through the Saudi Arabia Pediatric Hematology Oncology Society (SAPHOS) and the WhatsApp groups of the relevant pediatric and oncology–hematology departments. Participants interested in the study were provided with a link to the online questionnaire and asked to complete it independently.

### 2.4 Statistical analysis

Statistical analysis of the collected data was performed using the Statistical Package for the Social Sciences (SPSS) software (version 27; IBM Corp., Armonk, NY, USA). Frequencies and percentages were used to summarize qualitative variables, and the results were presented in tables or graphs. A chi-squared test was used to assess the association between physicians’ sociodemographic and professional characteristics and their views on AI in clinical practice. The Fisher–Freeman–Halton exact test was used whenever the expected cell count was less than 5. A *p*-value of < 0.05 was considered statistically significant.

## 3 Results

Table 1 describes the demographic characteristics and work environment of the 132 participants in this study. Overall, 50% of the participants were aged between 31 and 40 years, 30.3% were aged between 41 and 50 years, and 18.2% were older than 50 years. The distribution of participants by sex was relatively even, with 53% being male and 47% being female. Nationality data revealed that 62.9% of participants were Saudi nationals and 37.1% were non-Saudi. In total, 90% of the participants were specialists in both hematology and medical oncology. In terms of professional rank, 58.3% of the participants were consultants.

**Table 1:** Sociodemographic and work characteristics of the participating physicians.

Characteristic		*N* = 132	%
Age, years	<30	2	1.5%
	31–40	66	50.0%
	41–50	40	30.3%
	>50	24	18.2%
Sex	Male	70	53.0%
	Female	62	47.0%
Nationality	Saudi	83	62.9%
	Non-Saudi	49	37.1%
Subspecialty	Hematologist and oncologist	120	90.9%
	Oncologist	7	5.3%
	Hematologist	5	3.8%
Professional title	Consultant	77	58.3%
	Fellowship	26	19.7%
	Associate consultant	14	10.6%
	Registrar	9	6.8%
	Physician staff	6	4.5%
Work experience, years	<5	37	28.0%
	5–10	42	31.8%
	11–15	13	9.8%
	>15	40	30.3%
Place of work in Saudi Arabia	West	56	42.4%
	Central	39	29.5%
	East	22	16.7%
	South	11	8.3%
	North	4	3.0%

*N*, number.

Additionally, 10.6% of the participants were associate consultants, 19.7% were in fellowship training, 6.8% were registrars, and 4.5% were classified as staff physicians. Years of experience showed a relatively even distribution, with 28% having less than 5 years, 30.3% having more than 15 years, and 31.8% having 5–10 years of experience. The majority of the participants worked in the Western (42.4%) and Central (29.5%) regions.

Regarding physicians’ practices of AI in clinical practice, Table 2 shows that more than half (56.1%) of the physicians never used clinical AI decision support systems, while a minority (5.3%) used AI systems daily, and 8.3% used them at least once a week. However, diversity existed between participants who had not used any AI systems in the past year and those who had never used AI systems at all, as some participants had used AI decision support systems more than 1 year prior to the study. The majority of the physicians (68.9%) have never worked with AI-based decision support systems. Among those who used AI, 14.4% reported experiencing errors or accidents related to the use of AI. The majority (67.4%) of the physicians had unclear attitudes toward the use of clinical AI decision support systems, and only 5.3% of the physicians supported its use in clinical decision-making.

**Table 2:** The physicians’ practices of artificial intelligence in clinical practice.

Variable		*N* = 132	%
In the past year, how often have you used decision support clinical AI systems in practice?	Everyday	7	5.3%
	At least once a week	11	8.3%
	At least once a month	22	16.7%
	At least once every 6 months	10	7.6%
	Only once a year	8	6.1%
	Never	74	56.1%
Have any errors or accidents occurred while working with decision support clinical AI systems?	Never worked on decision support clinical AI systems	91	68.9%
	No	22	16.7%
	Yes	19	14.4%
What are patients’ attitudes toward the use of decision support clinical AI systems?	Support	7	5.3%
	Neutral	32	24.2%
	Oppose	4	3.0%
	Unclear	89	67.4%

AI, artificial intelligence; N, number.

The physicians’ attitudes toward AI integration into clinical practice were classified as positive in 98 (74.2%) participants. Table 3 shows the relationships between various demographic and professional factors and physicians’ attitudes. Physicians’ professional titles (*p* = 0.011) and work experience (*p* = 0.019) significantly impacted their attitudes toward AI integration. Registrars (4.1%) and resident physicians (2%) had significantly low positive attitudes, whereas fellows (23.5%) and associate consultants (12.2%) had moderately positive attitudes. Physicians with 11–15 years of experience had significantly low positive attitudes (5.1%). Physicians with less than 5 years of experience (30.6%) and those with 5–10 years of experience (32.7%) had moderately positive attitudes. However, these subgroup findings should be interpreted cautiously because some categories included relatively small numbers of participants. Age, gender, subspecialty, and workplace location were not significantly related to attitudes (all *p* > 0.05).

**Table 3:** Relationships between the attitudes of physicians toward artificial intelligence integration into clinical practice and their sociodemographic and work characteristics.

Variables		Attitude toward AI integration into clinical practice				*P*-value
		Negative *N* = 34 (25.8%)		Positive *N* = 98 (74.2%)		
Age, years	<30	1	2.9%	1	1.0%	0.803
	31–40	16	47.1%	50	51.0%	
	41–50	11	32.4%	29	29.6%	
	>50	6	17.6%	18	18.4%	
Sex	Female	17	50.0%	45	45.9%	0.681
	Male	17	50.0%	53	54.1%	
Subspecialty	Hematologist and oncologist	29	85.3%	91	92.9%	0.167
	Hematologist	3	8.8%	2	2.0%	
	Oncologist	2	5.9%	5	5.1%	
Professional title	Consultant	20	58.8%	57	58.2%	0.011*
	Fellowship	3	8.8%	23	23.5%	
	Associate consultant	2	5.9%	12	12.2%	
	Registrar	5	14.7%	4	4.1%	
	Physician staff	4	11.8%	2	2.0%	
Work experience, years	<5	7	20.6%	30	30.6%	0.019*
	5–10	10	29.4%	32	32.7%	
	11–15	8	23.5%	5	5.1%	
	>15	9	26.5%	31	31.6%	
Place of work in Saudi Arabia	Central	7	20.6%	32	32.7%	0.136
	East	9	26.5%	13	13.3%	
	North	1	2.9%	3	3.1%	
	South	5	14.7%	6	6.1%	
	West	12	35.3%	44	44.9%	

Others^#^ include registrars, fellowships, and physician staff. AI: artificial intelligence. The chi-squared tests and Fisher–Freeman–Halton exact tests were used. *Significant at *p* < 0.05.

**Figure 1:**
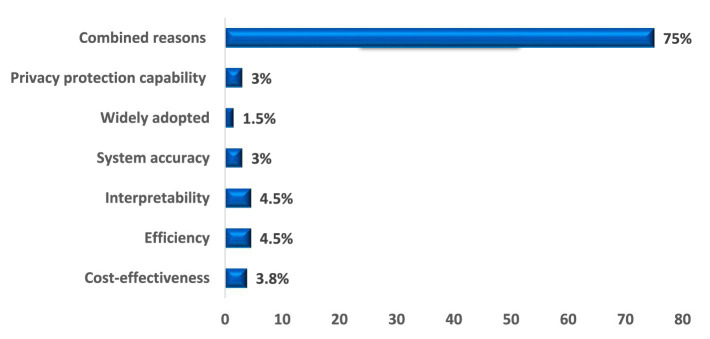
Determinants of physicians’ willingness to use clinical artificial intelligence in practice.

Figure 1 illustrates the key factors influencing physicians’ willingness to integrate AI into clinical practice. The majority of the physicians (75%) referred to multiple factors rather than a single factor for their willingness to use AI in clinical practice. This combination included efficiency, accuracy, cost-effectiveness, interpretability, data privacy, and widespread adoption.

**Figure 2:**
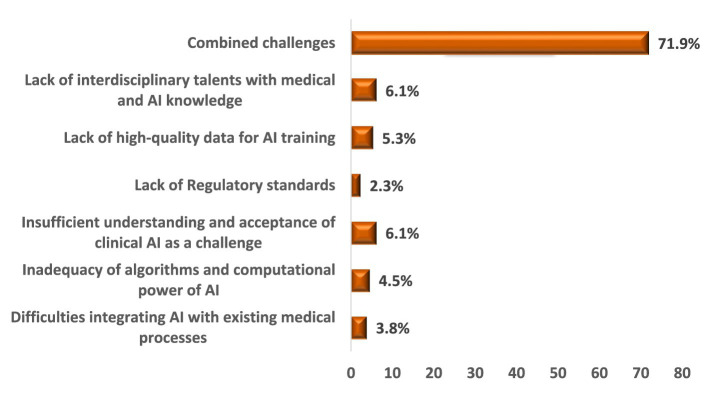
Challenges encountered during the development and integration of clinical artificial intelligence.

Figure 2 presents the challenges encountered during the development and integration of AI into clinical practice. The majority of the respondents (71.9%) reported multiple factors affecting AI integration rather than a single challenge. These limitations included a lack of interdisciplinary talent, lack of high-quality data for AI training, insufficient understanding and acceptance of AI, and weak regulatory frameworks, in addition to algorithmic and computational limitations.

**Figure 3:**
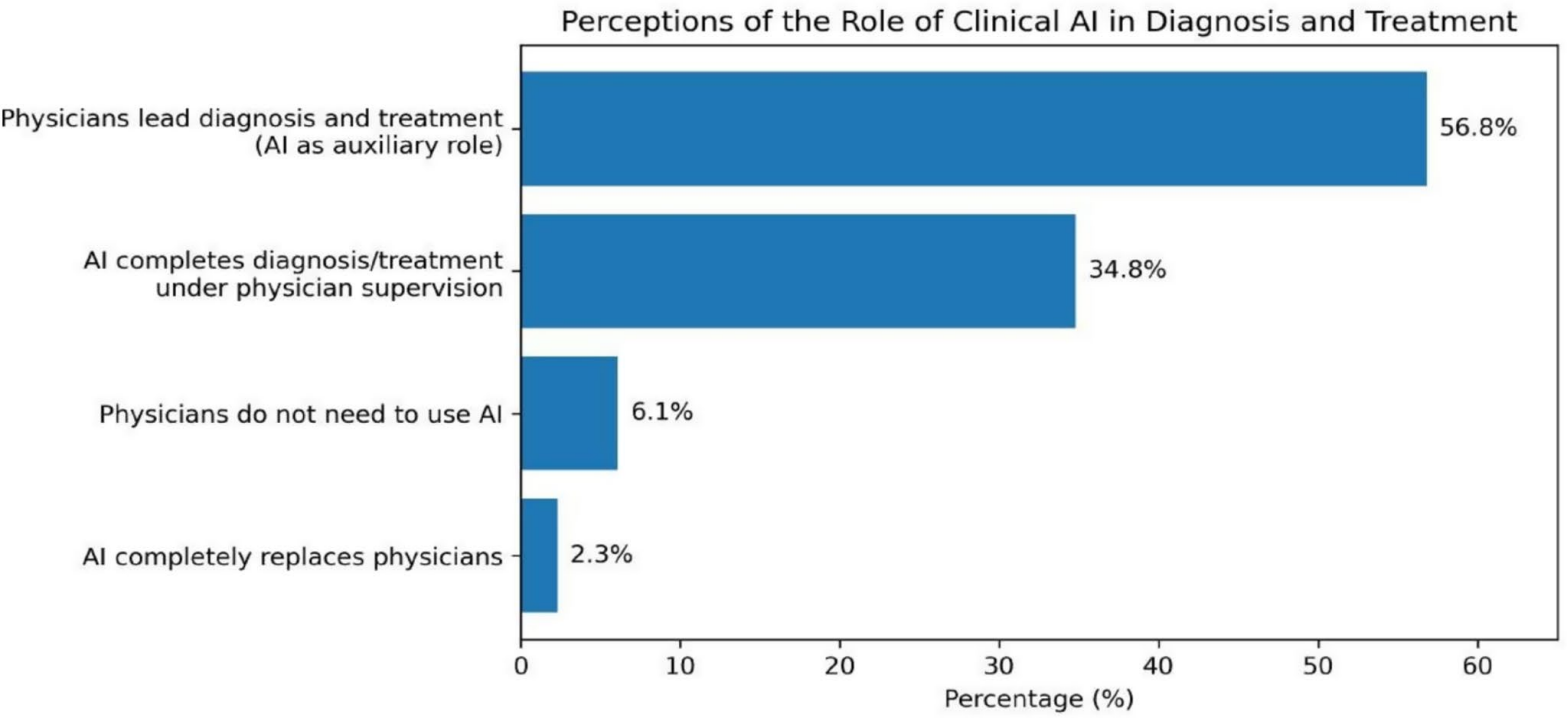
Participants’ perceptions of the relationship between physician and artificial intelligence (AI).

More than half (56.8%) of the respondents believed that human physicians should remain the primary decision-makers, with AI playing a supportive role in diagnosis and treatment decisions. Moreover, approximately one-third (34.8%) of the respondents believed that AI could take a more active role by independently performing tasks under physician oversight (Figure 3).

## 4 Discussion

The incorporation of artificial intelligence into the clinical practice of pediatric hematology–oncology physicians is rapidly evolving. Artificial intelligence has the potential to support clinical care in cancer and hematologic disorders by enhancing diagnostic processes, supporting treatment planning, and improving outcome prediction. The ongoing dialogue between AI developers and the healthcare professionals who will use these systems in the best interest of patients is of utmost importance to ensure their use in the most effective and ethical way within the daily clinical workflow to maximize benefits for patients (13, 14).

This study aimed to explore the opinions and concerns of physicians working in pediatric hematology–oncology clinics in Saudi Arabia regarding the use of AI. It also aimed to outline the potential benefits and identify challenges for the healthcare of children and adolescents with cancer and to map out the future implementation of AI in the healthcare system based on healthcare providers’ expectations.

Among pediatric hematology–oncology physicians surveyed in this study, 74.2% reported a positive view of AI in the clinical setting. Slightly more than half of the participants (56.1%) reported that they had never used clinical AI decision support systems, and the majority of the participants (68.9%) reported that they had never worked with AI-based decision support systems. The attitudes physicians hold toward the use of AI in clinical settings likely stem more from their perceptions and expectations than from direct experience with technology (15). However, our results should be interpreted with the caveat that they primarily reflect hypothetical acceptance and perceived readiness for AI rather than actual engagement with AI-powered clinical workflows. Our findings are generally positive regarding attitudes toward AI, consistent with prior studies on the adoption of AI in psychiatry, radiology, and pathology (16–18). A systematic review of the literature found that more than 60% of physicians and students had a generally positive but cautious view of AI (19). Similar studies have been conducted in Jeddah and other Middle Eastern countries. Although healthcare providers’ attitudes toward AI are very positive, they lack knowledge of AI (12, 20–22). However, a study conducted by Barakat et al. (23) found that many Saudi ophthalmologists believed that AI would reduce the demand for physicians needed in healthcare. In a German study, only 42.9% of physicians expressed a positive (or highly positive) view of AI in medicine, although most expected it to play a large role in the future of healthcare (24).

Attitudes of physicians toward AI integration in pediatric hematology–oncology are influenced by physicians’ professional titles and years of experience. Senior physicians, including fellows and associate consultants, exhibited a more positive attitude toward AI than registrars and resident physicians. Physicians with 11–15 years of experience had the least positive views, while those with less than 10 years of experience showed moderately positive views. These results can be influenced by a variety of factors, including exposure to AI, physicians’ confidence in evaluating health technology, and the perceived impact of AI on their current workflow. These findings remain to be explored in more detail, although they should be interpreted in light of the study’s sample distribution and the limited size of the subgroups. Several factors are perceived to influence physicians’ willingness to integrate AI into their practice. The majority of participants reported a combination of factors and did not identify a single key determinant. Participants identified perceived accuracy and efficiency, cost-effectiveness, and interpretability, as well as concerns about data privacy and security issues. The majority of the factors that affect the acceptance of AI were found to have either positive or negative effects; there is a balance between benefits and ethical or practical problems.

In addition, a number of barriers to the implementation of AI were perceived by the majority of the participants, including a lack of interdisciplinary skills and the use of low-quality data, a lack of familiarity with and the need for training in AI use, a variety of ethical, legal, organizational, and regulatory barriers, and a number of computational limitations (25, 26).

More than half of the participants surveyed believed that, in the future, physicians should be the primary decision-makers in patient care and that AI should support them. Consistent with previous studies (12, 15), the majority of the participants surveyed believed that future physicians should be the primary decision-makers in patient care, with AI serving a supportive role. This may reflect the view that human beings are better than computers at dealing with many aspects of care that require a more complex approach, such as accountability, transparency, and ethics (27). Several respondents supported a more autonomous role for AI in healthcare under the supervision of doctors. This suggests that there is scope for a “graded” introduction of AI to different clinical areas. A literature review on the impact of AI on empathy and compassion in person-centered care found that two strategies could help ensure AI had a positive influence on the doctor–patient relationship. First, AI could be used as an assistive tool and second, medical education would need to be adapted to include AI in several roles (28). The review highlighted the need for several strategies for the integration of AI into healthcare, as well as their implementation in a way that maintains the doctor’s central role while allowing AI to perform functions such as data analysis, pattern recognition, and clinical decision-making (29).

To conclude, the physicians’ views and anticipated behaviors regarding AI should not be confounded with their actual experience of AI in clinical applications. This is an important consideration when interpreting the results and when generalizing them to real-world practice settings.

## 5 Limitations

This study has several limitations. First, the study is cross-sectional, and it is not possible to establish any causal relationship between the variables in the study. The findings represent the viewpoints of the physicians at a single point in time and may change as technologies develop. Moreover, this study relies on self-reported data, which may be affected by biases such as recall and social desirability bias. In addition, the study participants were recruited through the SAPHOS and WhatsApp groups via a convenience sampling approach with voluntary participation, rather than through true random sampling. Hence, there may be a number of biases, including selection bias, and the findings may not be representative or generalizable to other groups. The study participants were recruited from different regions of Saudi Arabia; however, the sample size is relatively small, and the majority of participants were hematology–oncology specialists. Therefore, the findings may not be generalizable to other specialties in different healthcare settings. Furthermore, some professional subgroups were underrepresented; therefore, the findings may not be sufficient to compare the viewpoints of different physician subgroups. Some of the subgroup analyses had a relatively small number of participants; therefore, these findings should be treated with caution and are primarily exploratory in nature, with reduced statistical power to detect differences between subgroups.

Notably, while the majority of participants had little to no experience with AI decision support systems in clinical practice, their attitudes were likely influenced by growing awareness, clinical experience, and practice, as well as their own projections for the future use of AI in healthcare. Therefore, the findings are based on perceptions and anticipated attitudes that have not been implemented or evaluated in real clinical settings. A further limitation of the questionnaire was that it generally asked about participants’ attitudes to AI integration in a broad sense, rather than distinguishing among different clinical applications, such as AI-supported diagnosis and therapy planning, support during tumor board discussions, relapse management, or monitoring of patient treatment. The questionnaire used was adapted from an existing study. Extensive psychometric validation and formal reliability testing of the adapted questionnaire in the study population of interest were not conducted; therefore, the attitude measurement may not be as precise as intended. The study assessed participants’ perceived attitudes toward AI in healthcare rather than their knowledge, competency, or actual clinical outcomes associated with AI use.

## 6 Conclusion

The majority of pediatric hematology–oncology physicians in Saudi Arabia are positive about the use of AI in their practice. Professional titles and years of work experience played significant roles in the opinions expressed by the participants. Fellows and associate consultants were more positive than registrars and resident physicians. In addition, those with 11–15 years of work experience had less positive attitudes than their younger counterparts. The factors that influence physicians’ willingness to adopt AI include efficiency, accuracy, cost-effectiveness, interpretability, privacy, and the level of adoption in practice. The challenges of integrating AI into practice are numerous and multifaceted, including an insufficient number of interdisciplinary teams with AI expertise, a lack of high-quality data, limited clinician understanding and acceptance of AI, and computational challenges. More than half of the participants preferred that the AI serve as a support system for physicians, with the physician being the primary decision-maker.

The perceptions and attitudes of physicians regarding AI, as depicted in the findings above, are more influential than their actual clinical experience with AI. These findings can guide future implementation strategies for AI in clinical practice by aligning with physicians’ expectations and addressing issues related to training, governance, and system readiness.

## Data Availability

The original contributions presented in the study are included in the article/supplementary material, further inquiries can be directed to the corresponding authors.
